# Biochar–Clay–Compost Composite Materials for Sustainable Soil Management: Effects on Soil Biochemical Activity, Pore Architecture, and Water Retention

**DOI:** 10.3390/ma19183916

**Published:** 2026-09-15

**Authors:** Krzysztof Gondek, Edyta Jacak, Tomasz Głąb, Michał Kopeć, Monika Mierzwa-Hersztek, Agnieszka Baran, Jerzy Wieczorek

**Affiliations:** 1 Department of Agricultural and Environmental Chemistry, University of Agriculture in Krakow, Al. Mickiewicza 21, 31-120 Krakow, Poland; krzysztof.gondek@urk.edu.pl (K.G.); edyta.jacak@student.urk.edu.pl (E.J.); michal.kopec@urk.edu.pl (M.K.); agnieszka.baran@urk.edu.pl (A.B.); jerzy.wieczorek@urk.edu.pl (J.W.); 2 Department of Machinery Exploitation, Ergonomics and Production Processes, University of Agriculture in Krakow, ul. Balicka 116B, 31-149 Krakow, Poland; tomasz.glab@urk.edu.pl

**Keywords:** biochar, smectite–silica clay, compost-based composites, organic–mineral amendment materials, pore architecture, soil water retention, environmental materials, sustainable soil management

## Abstract

**Highlights:**

- Biochar, smectite–silica clay, and composts were evaluated as functional soil amendment materials.
- Biochar increased storage pore volume and water retention relative to the unfertilized control.
- Clay-containing materials gave the highest bulk density and permanent wilting point.
- Compost-derived composites balanced soil biological activity and physical performance.
- Organic–mineral composite materials showed potential for sustainable environmental applications.

**Abstract:**

The development of sustainable soil amendment materials derived from natural and recycled resources represents an important strategy for improving soil functionality and mitigating the impacts of land degradation and climate change. This pot study evaluated the effects of biochar (BC), poultry litter (PL), smectite-silica clay (SSC), and two composted combinations, C(PL+BC) and C(PL+BC+SSC), on soil biochemical activity, pore structure, and water retention. Seven treatments included an unfertilized control, mineral fertilization (MF), and MF combined with each amendment. The highest cumulative oxygen demand was recorded in MF+SSC (1.805 mg O_2_ g^−1^ DM), whereas the lowest occurred in MF+C(PL+BC) (1.356 mg O_2_ g^−1^ DM). Mineral fertilization produced the highest dehydrogenase activity. Compared with CTR, MF+BC had a larger volume of storage pores (0.5–50 μm) and produced the highest field capacity (0.385 cm^3^ cm^−3^) and available water content (0.265 cm^3^ cm^−3^). MF+SSC had the highest bulk density (1.309 g cm^−3^) and permanent wilting point (0.120 cm^3^ cm^−3^), but its available water content did not differ significantly from CTR. The first two principal components explained 58.0% of the variance and represented gradients associated with water retention and with bulk density and fine porosity. Among the tested materials, BC and C(PL+BC+SSC) provided the most balanced improvements in soil functionality by simultaneously enhancing water retention and maintaining moderate biological activity. The results indicate that biochar-, clay-, and compost-based amendment materials have potential for improving soil quality and supporting sustainable land management.

## 1. Introduction

Soil is a valuable, non-renewable resource for agroecosystems, and the health of these ecosystems has recently become a global concern. Healthy soil is a harmonious, highly complex matrix with good structure, optimal functionality, and effective buffering capacity. This type of soil ecosystem maintains a dynamic balance among all production factors [1,2]. Ongoing agricultural intensification and climate change increase the importance of implementing sustainable soil management strategies that improve soil functionality and resilience to environmental stress.

There is an urgent need to evaluate sustainable soil management strategies using functional indicators that measurably reflect soil fertility, health, and the ability to perform ecosystem functions. According to Drobnik et al. [3], numerous frameworks exist for soil quality assessments and evaluations, incorporating various forms of aggregation and indicators. These frameworks often indicate a deterioration in soil quality. It is estimated that degradation affects at least 24% of the world’s agricultural land, and projections indicate a further increase in this proportion in the coming years [4,5,6,7]. The causes of declining soil fertility are often linked to intensive agricultural practices, depletion of plant nutrients, a reduction in soil organic matter content, and disturbances in the ionic balance of the soil solution [2,3,8]. The issue of excessive use of mineral fertilizers, as well as increasingly frequent rainfall deficits resulting from dynamic climate change, is also significant for soil health. These factors significantly influence soil chemical properties, water availability, and microbial activity. Consequently, these elements shape the rate of biochemical processes, which in turn affects the circulation of elements in the environment [9].

In order to mitigate adverse changes in soil function, solutions are being sought that are based on the principles of sustainable soil resource management, which allow for maintaining agricultural productivity while reducing environmental pressure. In recent years, there has been a particular focus on organic and mineral materials used as soil amendments that can improve soil physicochemical and biological properties and increase organic matter stability. This group of materials includes biochar, natural fertilizers (e.g., poultry litter), smectite-silica clays, and composts produced using these materials, considered biologically stabilized materials [10,11,12]. Previous studies have mainly focused on the effects of organic materials. However, knowledge regarding the effects of organic–mineral amendments and their capacity to concurrently influence soil biological and physical properties remains limited.

Thanks to its high porosity, large specific surface area, and relative resistance to mineralization, biochar (BC) increases soil organic carbon content, improves water retention, and promotes the sorption of nutrients and pollutants. Furthermore, BC improves soil properties, creating favorable conditions for the growth and development of soil microorganisms [13]. Its mechanisms of action include improving pore structure, regulating pH, increasing water and nutrient availability, and reducing the toxicity of substances harmful to microorganisms [14].

Poultry litter (PL) is a rich source of macro- and micronutrients, as well as readily available organic matter. However, its direct application can lead to ionic imbalances and nutrient losses [15,16]. Consequently, the stabilization of PL through composting or mixing with sorbent materials is being studied more extensively, as this reduces environmental risks [17].

Clay minerals, including smectite-silica clays, play a significant role in shaping the soil sorption complex and limiting nutrient leaching due to their high cation exchange capacity and water-binding ability. Using them in combination with organic materials promotes the formation of durable mineral-organic complexes that increase soil carbon stability and positively influence soil aggregate structure. Nizbat et al. [18] reported that clay minerals, particularly smectites, may contribute to the stabilization of organic matter in agricultural soils. The high cation exchange capacity, large specific surface area, and ability to form electrostatic bonds and complexes with organic compounds of smectites enable their effectiveness in protecting organic carbon from microbial mineralization. The authors emphasize that combining organic additives with the clay fraction promotes the formation of stable mineral-organic complexes, which may contribute to greater protection and retention of organic carbon in soil, as well as improved structural stability and biogeochemical functioning. Composts produced from biochar, poultry litter, and clay minerals, including smectite-silica clay, constitute a form of biologically stabilized matter that is characterized by lower susceptibility to rapid decomposition and a more controlled release of nutrients [19]. These composts impact soil functional indicators, such as pH, nutrient content, water-holding capacity, and soil microorganism activity.

Most previous studies have examined individual amendments or have focused primarily on either biological or physical soil responses. Direct, side-by-side comparisons of organic, mineral, and organic–mineral composite amendments under the same experimental conditions, integrating both biological and physical soil responses, remain limited. It is therefore unclear whether improvements in microbial activity are accompanied by favourable changes in pore structure and water retention, or whether some amendments improve one group of properties at the expense of another. This distinction is important because greater total water retention does not necessarily translate into greater water availability to plants.

We hypothesized that the tested amendments would produce distinct responses rather than a uniform improvement in all soil properties. Biochar and composts containing biochar were expected to increase the volume of pores associated with plant-available water. Smectite–silica clay was expected to increase water retention mainly through an increase in finer pores, whereas poultry litter was expected to stimulate biochemical activity by supplying readily available nutrients and organic substrates. The objective of this study was to directly compare biochar, poultry litter, smectite–silica clay, compost produced from poultry litter and biochar, and compost produced from poultry litter, biochar, and smectite–silica clay, all applied with mineral fertilization, with respect to soil chemical properties, dehydrogenase and respiratory activity, pore size distribution, water retention characteristics, and ryegrass biomass.

## 2. Materials and Methods

### 2.1. Characteristics of Soil

The soil used for the pot experiment was collected from a depth of 0–20 cm in southern Poland. The soil had a particle size distribution of sandy loam (sand 320 g kg^−1^, silt 490 g kg^−1^, and clay 190 g kg^−1^). The pH value in the soil water suspension was 7.03, and the electrical conductivity (EC) was 354 µS cm^−1^. The total nitrogen (N_tot_) and carbon (C_org_) contents were 1.49 and 13.65 g kg^−1^ DM, respectively, and the total copper (Cu), cadmium (Cd), lead (Pb), and zinc (Zn) contents were 18.8, 1.67, 71.0, and 243 mg kg^−1^ DM, respectively.

### 2.2. Soil Amendments

The study used biochar (BC), poultry litter (PL), smectite-silica clay (SSC), compost made from litter and biochar (C(PL+BC)), and compost made from litter and biochar with the addition of smectite-silica clay (C(PL+BC+SSC)). Poultry litter came from a domestic guinea fowl farm. The birds were reared in a peat environment at a stocking density of 6 birds m^2^. The animals were fed commercial feed mixtures. Biochar was produced from coniferous wood waste at a temperature of 450 °C [20]. Smectite-silica clay was sourced from the Dylągówka-Zapady deposit [21]. The composts were produced under controlled laboratory conditions at the experimental station of the Faculty of Agriculture and Economics at the University of Agriculture in Krakow (URK). The composition of the biomass for composting was 50% PL and 50% BC. SSC was incorporated into the aforementioned biomass at a rate of 0.5% (relative to the dry matter of the mixture) [22]. Detailed information regarding the production of the composts and their characteristics has been described in a previous publication by Gondek et al. [22]. Selected chemical properties of the materials used in the study are presented in Table 1.

### 2.3. Pot Experiment and Sampling

The pot experiment was conducted in the URK vegetation hall in Mydlniki, Krakow. The experiment was set up in PVC pots measuring 235 mm in diameter and 240 mm in height. Each pot was filled with 8 kg of air-dried, 10-mm-sieved soil. The experimental design included 7 treatments, each carried out in 3 replicates: CTR—soil without additions, MF—soil with added mineral salts (NPK), MF+BC—soil with added mineral salts and biochar, MF+PL—soil with added mineral salts and poultry litter, MF+SSC—soil with added mineral salts and smectite-silica clay, MF+C(PL+BC)—soil with added mineral salts and compost made from poultry litter and biochar, and MF+C(PL+BC+SSC)—soil with added mineral salts and compost from poultry litter and biochar enriched with smectite-silica clay. The experimental design included two control treatments, namely CTR and MF. To create comparable conditions in each treatment (except for CTR), chemically pure mineral salts were used: KH_2_PO_4_, KNO_3_, and NH_4_NO_3_. Nutrients were applied to the soil at the following rates: 0.20 g N kg^−1^ DM soil, 0.10 g P kg^−1^ DM soil, and 0.25 g K kg^−1^ DM soil. The application rate of PL, BC, SSC, and compost (C) was 0.5% of soil dry matter, i.e., 40 g of additive pot^−1^, which corresponded approximately to a rate of 15 t of material ha^−1^.

After mixing the additives with the soil, the mixture was moistened to 30% of its maximum water-holding capacity. Then, it was left for 12 days. After this period, perennial ryegrass (Grasslands Nui m.k. C1) seeds were sown at a rate of 3.5 g pot^−1^. Throughout the experiment, the plants were watered with deionized water until the soil reached 60% of its maximum water-holding capacity. During the growing season (112 days), two harvests were collected on 3 July and 18 August 2025. After the growing season ended, soil samples were collected from each pot using a soil probe across the entire cross-section of the pot. The soil samples were then 2-mm-sieved. A portion of the soil sample was cooled to 4 °C for transport to the laboratory for biochemical analysis, including determination of respiratory and dehydrogenase activities. The rest of the sample was dried at room temperature (~25 °C) for chemical analysis.

To characterize soil retention, two intact cores were collected from each pot in 100-cm^−3^ stainless steel Kopecky cylinders (5.02 cm diameter and 5.05 cm length). The cylinders had sharp edges to facilitate insertion into the soil and brass caps to protect the samples from moisture loss over an extended period. This allowed the samples to retain their natural structure and moisture content. The cylinders were inserted vertically into a 5–10 cm layer of soil. The two cores were subsamples of the same pot. Their measurements were averaged before statistical analysis and curve fitting, so the pot was the experimental unit. Next, the plant roots were removed from the soil sample and thoroughly washed with deionized water. The biomass of the aboveground parts and roots was dried to a constant weight at 60 °C in a natural convection oven. After drying, the biomass was weighed.

### 2.4. Chemical Analyses

The following parameters were determined in the soil: pH by the potentiometric method in a soil-water suspension (1:2.5), and electrical conductivity (EC) by conductometry. The nitrogen content was determined using the Kjeldahl method after reducing N-NO_3_ with Devarda’s alloy. The organic carbon content was determined according to the Tyurin method. The sum of exchangeable alkaline cations (S) and hydrolytic acidity (Hh) was determined by Kappen’s method, as follows: (S) after 1 h extraction with 0.1 mol dm^−3^ HCl; (Hh) after 1 h extraction with 1 mol dm^−3^ CH_3_COONa. Total exchangeable cations (T) and saturation with alkaline cations of the sorption complex (V) were calculated from the following relationships:

(1) T = Hh + S

(2) V(%) = (S/T) × 100

### 2.5. Biochemical Analyses

Dehydrogenase activity was determined by measuring the conversion of the colorless, water-soluble compound 2,3,5-triphenyltetrazolium chloride (TTC) into the water-insoluble compound 1,3,5-triphenylformazan (TPF). Samples were incubated at 37 ± 2 °C for 24 h. Enzymatic dehydrogenase activity was determined colorimetrically using a Beckman DU 640 spectrophotometer (Fullerton, CA, USA) at a wavelength of 485 nm [23].

The assessment of soil respiratory activity was conducted employing the manometric method with Oxitop^®^ Control measuring equipment (WTW GmbH, Weilheim, Germany), in accordance with ISO 14855-1:2012 [24]. The manometric measurement of soil respiratory activity entailed the continuous monitoring of pressure changes within closed vessels. These pressure changes are proportional to the amount of oxygen consumed by the sample due to microbial processes occurring within it [25]. The respiratory activity measurement duration was 7 days and included a lag phase to stabilize conditions and the period of actual respiratory activity measurements. Pressure changes were automatically recorded every 60 min. The resulting CO_2_, equivalent to the oxygen utilized by the microorganisms, was absorbed by a 1 mol dm^−3^ NaOH solution present in the vessels. During the assay, the measuring vessels were placed in a thermostatic cabinet designed to maintain a constant temperature of 25.0 °C (± 0.1 °C). Measurement data were transmitted to the controller via an infrared interface and then to a computer using the Achat OC software(version 3.2.0.0.). Respiratory activity in 100 g DM samples was calculated using the ideal gas equation and estimated to 0.001 mg O_2_ g^−1^ h^−1^ according to the formula given below. The analyses were performed in triplicate. The results are presented as functions of 1°, where “y” is the oxygen demand in mg O_2_ g^−1^ of soil, and “x” is time expressed in hours (h). The lag phase was considered, which in this study lasted 1 day (24 h) and was determined based on the onset of activity in the control sample.

(3) $$RA=\frac{MO_{2}}{R\times T}\times \frac{v_{f_{r}}}{mB_{t}}\times \left|∆p\right|[mg{\;O}_{2}\times (g\times h{)}^{-1}]$$

where:

RA—respiratory activity;

MO_2_—molar mass O_2_ (31.999 g mol^−1^);

R—ideal gas constant; 83.14 L hPa (K mol)^−1^;

T—temperature (K);

mBt—dry matter of composted material (kg);

|Δp|—pressure changes (hPa);

Vfr—free gas volume (dm^3^).

### 2.6. Measurements of Soil Physical Parameters

Soil water retention properties were determined using a pressure plate apparatus (Soil Moisture Equipment Corp., Santa Barbara, CA, USA), with three independent pots analyzed for each treatment [26]. Prior to measurement, soil samples were saturated for 24 h and subsequently subjected to equilibration at 8 matric potentials: −4, −10, −33, −100, −200, −500, −1000, and −1500 kPa. The measured water retention data were described using the van Genuchten–Mualem model [27,28], with model parameters estimated through nonlinear least-squares regression.

Soil physical and hydraulic indicators were calculated from the measurements and soil water retention curves. Total porosity (TP) was derived from soil particle density (PB) and dry bulk density (BD). Field capacity (FC) was defined as the volumetric water content at a matric potential of −10 kPa, whereas the permanent wilting point (PWP) corresponded to the water content at −1500 kPa. Relative field capacity (RFC) was computed as the ratio of FC to TP following Reynolds et al. [29]. The slope (S) at the inflection point of the water retention curve was also determined. Available water content (AWC) was calculated as the difference between FC and PWP, while productive water content (PWC) was defined as the difference between FC and the water content measured at −500 kPa.

Pore size distribution (PSD) was estimated from the modeled soil water retention curves in accordance with Ahuja et al. [30]. Pores were classified into five functional groups based on equivalent pore diameter: bonding pores (<0.005 μm), residual pores (0.005–0.5 μm), storage pores (0.5–50 μm), transmission pores (50–500 μm), and fissures (>500 μm), following the classification proposed by Greenland [31].

### 2.7. Statistical Analyses

Data were analyzed using the statistical package Statistica, version 13.1 (StatSoft Inc., Tulsa, OK, USA) and Microsoft Excel 2016. For the physical and water retention variables, one mean per pot was used as one observation. Residual normality was checked with the Shapiro–Wilk test and homogeneity of variance with Levene’s test. One-way ANOVA was used to identify overall significant differences between the treatments. When significant differences were observed, treatment means were separated using Tukey’s HSD test, with a level of significance of *p* ≤ 0.05. ANOVA results (F, degrees of freedom, and *p*) and assumption checks for the physical and water retention variables are provided in Table S1. Treatment groups were compared with each other and with the control. Principal component analysis (PCA) was performed in Statistica v. 13.3 to explore relationships among soil physical, chemical, and biological properties. The analysis was based on the correlation matrix of standardized variables to account for differences in measurement units. The number of principal components was determined using the Kaiser criterion (eigenvalues > 1) and verified by inspecting the scree plot. Based on these criteria, the first two principal components were retained for further interpretation. Variable loadings were then used to identify the contribution of each variable to the respective principal component. Only variables with absolute loadings ≥ |0.60| were retained and emphasized for interpretation because they were considered to contribute strongly to the respective principal component.

Soil water retention curves were described using the van Genuchten model [27,28]. The relationship between volumetric water content (*θ*) and matric potential (*h*) and the magnitude of matric suction (*h* ≥ 0, expressed in kPa) was expressed as:

(4) $$\theta \left(h\right)=\theta_{r}+\frac{\theta_{s}-\theta_{r}}{{\left(1+(\alpha h{)}^{n}\right)}^{m}}$$

where: *θr_r_* and *θs* are the residual and saturated volumetric water contents (cm^3^ cm^−3^), respectively, *α* is a scale parameter related to the inverse of air-entry suction (kPa^−1^), and *n* is a dimensionless shape parameter. The model was fitted separately to the mean retention curve of each pot. *θr* was fixed at 0 cm^3^ cm^−3^ and *θs* was fixed at the TP of the same pot. Only *α* and *n* were estimated by unweighted nonlinear least-squares regression, and m was calculated as 1 − 1/*n*. Each fit included all eight measured matric potentials. The fitted lines in Figure (Section 3.4) represent the means of the nine fitted curves at each suction. Model performance was evaluated using the coefficient of determination (R2) and the root mean square error (RMSE). 

## 3. Results

### 3.1. Chemical Parameters of Soil After Different Amendment Treatments

The pH values ranged from 6.94 to 7.25 (Table 2). The highest value was found in the CTR control treatment (7.25), and the lowest values were recorded in the MF (6.95) and MF+C (PL+BC+SSC) (6.94) treatments. Applying mineral fertilization significantly decreased the pH values compared to the control. The incorporation of biochar into the soil (MF+BC) led to a substantial decrease in the acidifying potential of mineral fertilizer. No significant variation in pH (H_2_O) values was observed in the soil of the remaining treatments.

Soil salinity levels in the individual treatments did not differ significantly, and electrical conductivity (EC) values ranged from 128 to 191 µS cm^−1^ (Table 2). While the differences in EC values were not statistically significant, they were lower in the MF and MF+BC treatments and higher in the treatments with compost and the MF+PL and MF+SSC mixtures than in the CTR treatment.

The organic carbon content (C_org_) ranged from 12.2 to 14.6 g kg^−1^ DM and did not differ significantly among soils from individual treatments. The highest C_org_ values were recorded in the MF+PL and MF soils (14.6 and 14.5 g kg^−1^ DM, respectively), while the lowest values were found in the MF+SSC and CTR soils (12.2 and 12.4 g kg^−1^ DM, respectively). These results suggest that introducing organic materials did not significantly increase the C_org_ content in the soil. Total nitrogen (N_tot_) in most soil treatments was similar, ranging from 1.41 to 1.49 g kg^−1^ DM (Table 2). However, the soil from the MF+C(PL+BC+SSC) treatment had a significantly higher N_tot_ content compared to the other treatments, amounting to 1.64 g kg^−1^ DM.

These findings suggest that the implemented treatments significantly impacted the characteristics of the soil sorption complex (Table 2). The CTR treatment exhibited the highest value of the sum of basic cations (S), which was recorded at 96.9 mmol(+) kg^−1^ DM, while the lowest value was observed in the MF+BC treatment. The absence of a clear increase in the S value of the soil following the application of organic materials suggests that their impact on the pool of exchangeable cations was constrained in the immediate term.

The hydrolytic acidity (Hh) was highest in the MF treatment (22.1 mmol(+) kg^−1^ DM), which indicated a potential acidifying effect of mineral fertilization, particularly nitrogen-based fertilizers. Soil from the CTR treatment had the lowest Hh value (15.1 mmol(+) kg^−1^ DM), indicating a higher degree of saturation of the sorption complex with bases (V). The addition of organic amendments (biochar, litter, compost) limited the increase in Hh values compared to MF alone, which may indicate a buffering effect.

Sorption capacity (T) was generally stable (Table 2). Significantly, the lowest T value was found in the MF+BC treatment. This may indicate that, depending on its physicochemical properties, the biochar used did not significantly increase the soil’s sorption capacity during the study period. The highest T value was obtained in the MF+C(PL+BC+SSC) variant, suggesting a beneficial effect of combining organic matter with the clay fraction on the sorption complex structure through stable organic-mineral complex formation.

The degree of saturation of the sorption complex with bases (V) was significantly the highest in the CTR soil (86.6%). In contrast, V values were lower (80.5–82.8%) in all treatments amended with the tested mixtures and in the MF treatment. The decline in V values in MF was primarily attributable to an increase in Hh values, while T values remained relatively stable. This indicates that, despite maintaining a comparable T value, the proportion of acid cations (Hh) in the sorption complex increased under the influence of mineral fertilization.

### 3.2. Dehydrogenase Activity of Soil After Different Amendment Treatments

**Figure 1:**
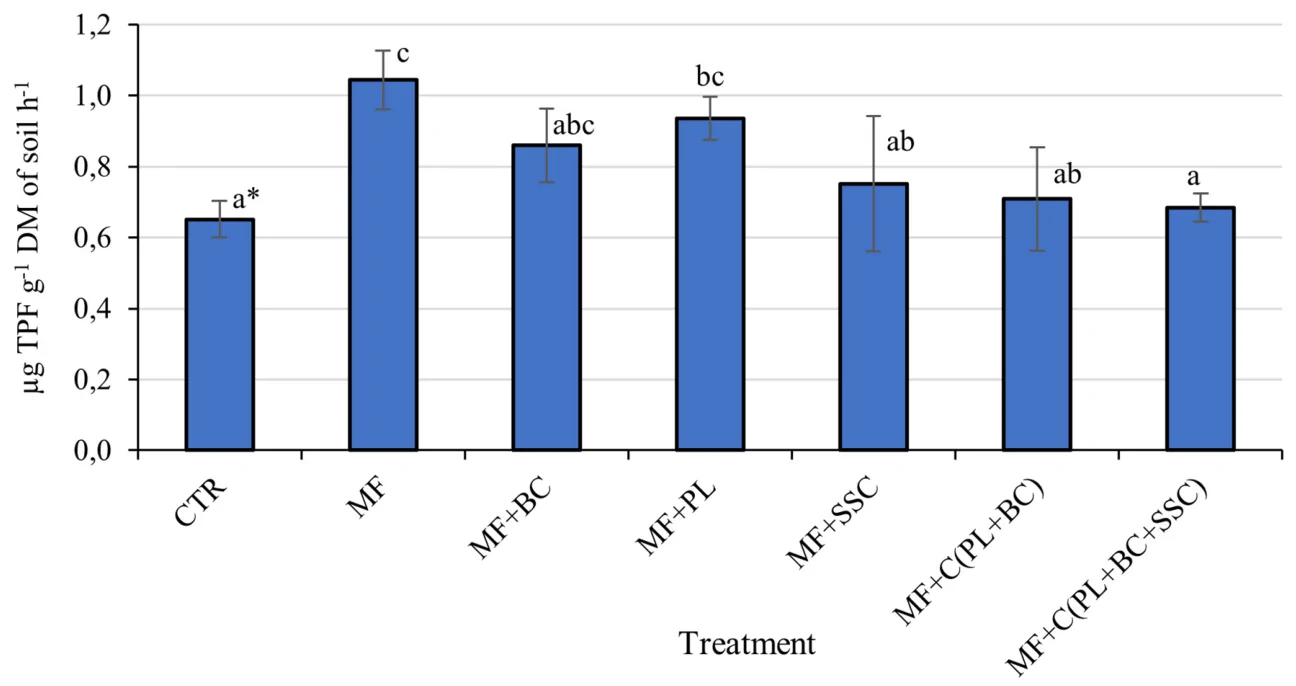
Dehydrogenase activity of soil after different amendment treatments, ± standard deviation, n = 3. * Different letters indicate a significant difference at *p* ≤ 0.05 according to Tukey’s HSD test. CTR—control soil; MF—soil with mineral fertilizers; MF+BC—soil with MF and biochar; MF+PL—soil with MF and poultry manure; MF+SSC—soil with MF and smectite–silica clay; MF+C(PL+BC)—soil with MF and compost made from poultry manure and biochar; MF+C(PL+BC+SSC)—soil with MF and compost made from poultry manure and biochar, with the addition of smectite–silica clay.

Dehydrogenase activity (DhA) serves as an indicator of the vitality and metabolic activity of soil microorganisms, which are associated with the oxidation of organic compounds in the soil. Our study findings indicate that the MF treatment exhibited the highest DhA, suggesting that enhanced nutrient availability fostered favorable conditions for soil microbial growth and activity (Figure 1).

This activity was significantly higher than in the CTR treatment, in which the lowest DhA values were recorded. The DHA in the MF+BC and MF+PL treatments was slightly lower than in the MF treatment but higher than in the CTR treatment. This finding suggests that organic amendments also promoted microbial activity, although this effect was not more pronounced than that observed in the MF treatment. The MF+SSC, MF+C(PL+BC), and MF+C(PL+BC+SSC) treatments exhibited lower, more variable DHA. This may result from differences in the availability of readily degradable organic substances, as well as the influence of mineral additives on soil microflora. The wide confidence intervals in some variants indicate variability in the response of microorganisms to the applied amendments. This variability may be attributable to differing rates of organic matter mineralization and varying environmental conditions within the soil.

### 3.3. Respiratory Activity of Soil After Different Amendment Treatments

The application of organic and mineral amendments significantly affected soil respiratory activity (RA), suggesting their potential influence on microbial processes and carbon cycling in soil. The high values of the coefficient of determination (R^2^ = 0.955–0.994) indicate a stable course of respiration processes during the analyzed incubation period, which is typical for soils with stabilized microbial activity (Table 3). The highest cumulative oxygen consumption was recorded in the MF+SSC treatment, indicating enhanced microbial activity and/or more intensive mineralization processes. Although SSC does not provide an easily mineralizable carbon source, its presence may have modified the physicochemical environment of the soil, for example, by increasing the sorption surface and water retention capacity (Table 4 and Table 5). Such changes may have altered the availability and accessibility of organic substrates and nutrients, as well as the habitat conditions for soil microorganisms, potentially affecting the decomposition of native soil organic matter. Therefore, the elevated respiration observed in the MF+SSC treatment may reflect indirect effects of SSC on microbial activity and carbon turnover rather than mineralization of carbon derived directly from the amendment. However, these mechanisms should be considered potential explanations, as the applied respiration measurements did not allow the carbon derived from the amendment to be distinguished from native soil organic carbon. Slightly lower soil respiratory activity (value of the directional coefficient “a”) was also observed in the MF+PL treatment. This response may be associated with the greater availability of nutrients and organic substrates that can stimulate microbial activity. The observed variations among treatments suggest differences in substrate availability and mineralization dynamics; however, the specific sources of respired carbon cannot be identified based on respiration measurements alone.

The lag phase in respiratory activity analysis, as determined in our own study, was relatively brief in all treatments, indicating that microbiological processes were still ongoing in the soil. The calculated trend lines in individual treatments exhibited slight differences in the parameters of the 1° equation. The “b” coefficient attained its maximum value for the MF+SSC treatment, with a measurement of 0.2816 mg O_2_ g^−1^ DM of soil. The lowest recorded value of this coefficient was observed for the MF+C(PL+BC+SSC) treatment. However, the addition of BC generally resulted in a reduction in the level of respiratory activity during the lag phase (state after 24 h of analysis).

The slope of the trend line (“a”) was also highest in the MF+SSC treatment (0.0129 mg O_2_ g^−1^ h^−1^), while values for the remaining treatments ranged from 0.0092 to 0.0118 mg O_2_ g^−1^ h^−1^. Consequently, the unit increase in oxygen consumption in MF+SSC was approximately 1.09–1.40 times greater than in the other treatments. These trends were reflected in the cumulative oxygen uptake measured after 7 days, which was highest in MF+SSC and significantly greater than in MF+C(PL+BC).

The lower respiration observed in biochar-containing treatments may reflect the lower biodegradability of biochar-derived carbon and/or reduced accessibility of organic substrates to microorganisms. However, because oxygen consumption was not partitioned between amendment-derived and native soil organic carbon, these results do not provide direct evidence of carbon stabilization or sequestration. Therefore, the observed differences in respiration should be interpreted as indicators of altered microbial activity and carbon turnover rather than direct measures of carbon preservation in soil.

### 3.4. Effect of Soil Amendment Application on Soil Pore Characteristics and Soil Water Retention Characteristics

**Figure 2:**
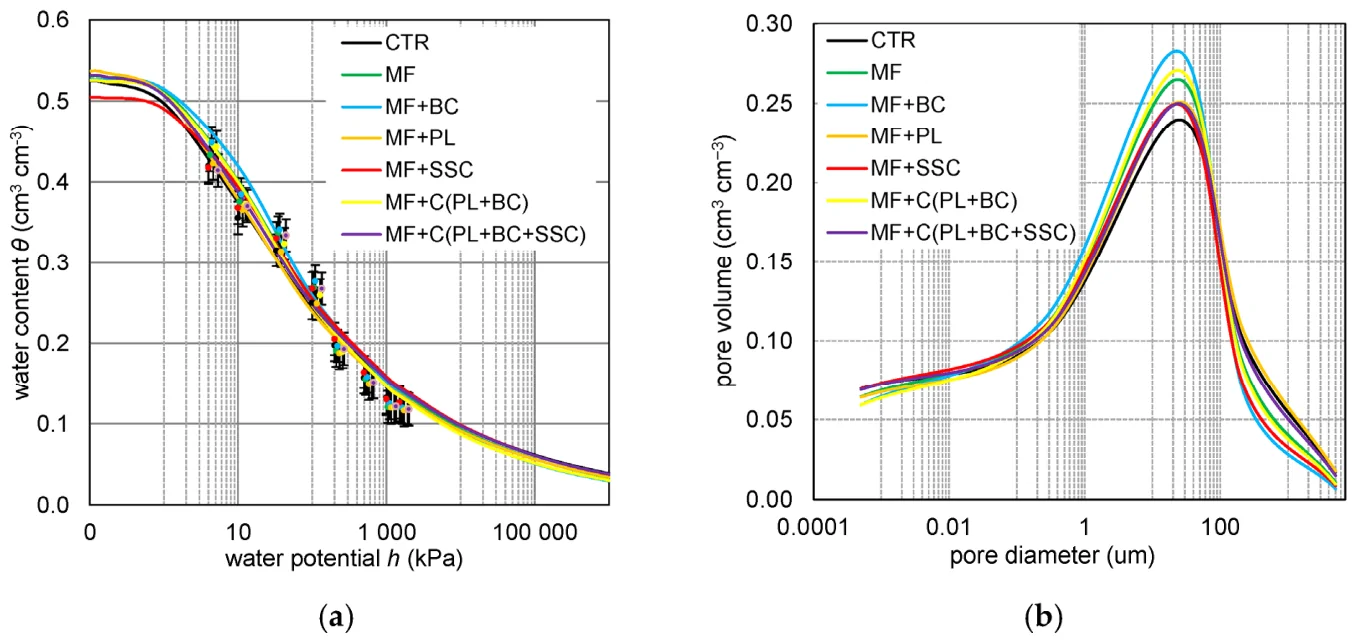
Soil water retention curves (**a**) and pore size distribution (**b**) after different soil amendment treatments. In panel (**a**), points represent mean measured water contents, and error bars indicate ± standard deviation (SD). CTR—control soil; MF—soil with mineral fertilizers; MF+BC—soil with MF and biochar; MF+PL—soil with MF and poultry manure; MF+SSC—soil with MF and smectite–silica clay; MF+C(PL+BC)—soil with MF and compost made from poultry manure and biochar; MF+C(PL+BC+SSC)—soil with MF and compost made from poultry manure and biochar, with the addition of smectite–silica clay.

Bulk density ranged from 1.220 to 1.309 g cm^−3^ (Table 4). MF+SSC had a significantly higher bulk density than all other treatments. Total porosity, calculated from bulk density and particle density, ranged from 0.506 to 0.540 cm^3^ cm^−3^ (Table 5) and was significantly lower in MF+SSC than in the other treatments. The pore size distributions derived from the fitted water retention curves are shown in Figure 2a. MF+BC had the largest volume of storage pores (0.5–50 μm), whereas CTR had the lowest. Treatments containing smectite-silica clay had relatively large volumes of the finest pores (<0.005 μm) (Table 5).

The volume of pores < 0.005 μm ranged from 0.059 to 0.070 cm^3^ cm^−3^ (Table 5). CTR, MF+SSC, and MF+C(PL+BC+SSC) had the highest values, whereas MF+BC and MF+C(PL+BC) had the lowest. For pores of 0.005–0.5 μm, MF+BC had a significantly larger volume than MF+PL, with other treatments intermediate. Storage pores of 0.5–50 μm accounted for the largest share of total porosity, with volumes from 0.240 to 0.282 cm^3^ cm^−3^ (Table 5). MF+BC had a significantly larger volume than CTR. No significant treatment differences occurred for transmission pores of 50–500 μm or fissures >500 μm.

The water retention curves differed mainly at less negative matric potentials (Figure 2b). MF+BC generally retained more water than CTR near field capacity. At more negative potentials, treatment differences were smaller, although MF+SSC had the highest water content at the permanent wilting point. The slopes at the inflection point were highest in MF+BC and lowest in MF+SSC (Table 4). Field capacity ranged from 0.356 to 0.385 cm^3^ cm^−3^. MF+BC had the highest value and differed significantly from CTR, whereas the remaining treatments were intermediate (Table 4). Permanent wilting point ranged from 0.111 to 0.120 cm^3^ cm^−3^. MF+SSC had the highest value and differed significantly from MF+PL but not from the other treatments.

Available water content ranged from 0.234 to 0.265 cm^3^ cm^−3^. MF+BC and MF+C(PL+BC+SSC) had the highest values, and CTR had the lowest (Table 4). Productive water content followed a similar pattern. The slope at the inflection point ranged from 0.0645 in MF+SSC to 0.0737 in MF+BC. Relative field capacity ranged from 0.676 to 0.731 and was highest in MF+BC and MF+SSC.

### 3.5. Ryegrass Biomass

**Figure 3:**
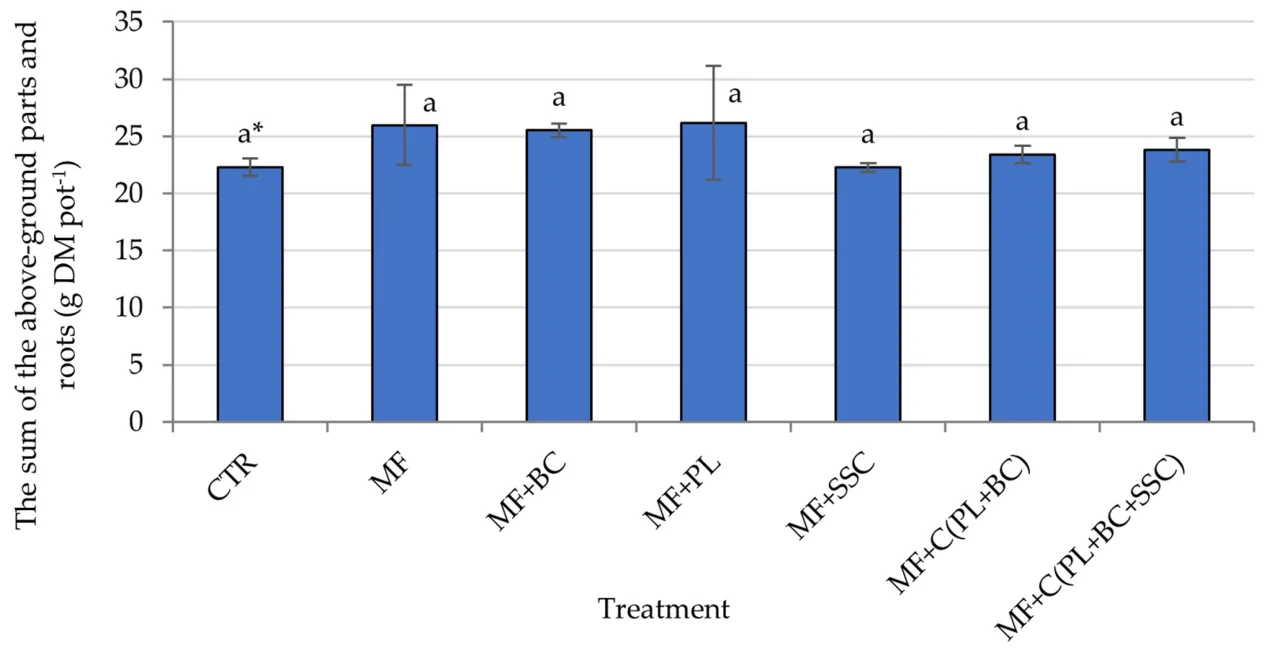
Total biomass of ryegrass collected in individual treatments, ±standard deviation, n = 3. * Different letters indicate a significant difference at *p* ≤ 0.05 according to Tukey’s HSD test. CTR—control soil; MF—soil with mineral fertilizers; MF+BC—soil with MF and biochar; MF+PL—soil with MF and poultry manure; MF+SSC—soil with MF and smectite–silica clay; MF+C(PL+BC)—soil with MF and compost made from poultry manure and biochar; MF+C(PL+BC+SSC)—soil with MF and compost made from poultry manure and biochar, with the addition of smectite–silica clay.

Figure 3 shows the total biomass of the aboveground parts and roots for each experimental treatment. The CTR treatment exhibited the lowest biomass, suggesting that the soil’s productivity is severely limited in the absence of external inputs such as mineral fertilizers or organic or mineral materials. The application of mineral fertilization (MF) resulted in a marked, more than twofold increase in biomass, indicating that nutrient availability may be an important factor influencing plant biomass production. The incorporation of biochar (MF+BC) into the soil resulted in the maintenance of the amount of harvested biomass at a level comparable to that of MF (88.35 g DM pot^−1^), indicating that, in the short term, its impact on the quantity of plant biomass produced was stabilizing. The MF+PL treatment yielded the greatest amount of ryegrass biomass. This observation suggests a potential positive impact of the introduced nitrogen and organic matter on the soil’s nutrient content and biological activity. In the MF+SSC treatment, a lower amount of ryegrass biomass (64.89 g DM pot^−1^) was recorded compared to other fertilized treatments. This suggests that the mineral component, when applied in isolation and without an additional source of organic matter, did not contribute to increased productivity. It is plausible that this may have temporarily limited the availability of plant nutrients. In the MF+C(PL+BC) treatment, the amount of harvested biomass was 80.93 g DM pot^−1^, while in the MF+C(PL+BC+SSC) treatment, the amount of harvested biomass was lower at 77.43 g DM pot^−1^. In both cases, the ryegrass biomass collected was greater than in the CTR, but did not exceed the amount of biomass harvested in the MF+PL treatment.

### 3.6. Principal Component Analysis

**Figure 4:**
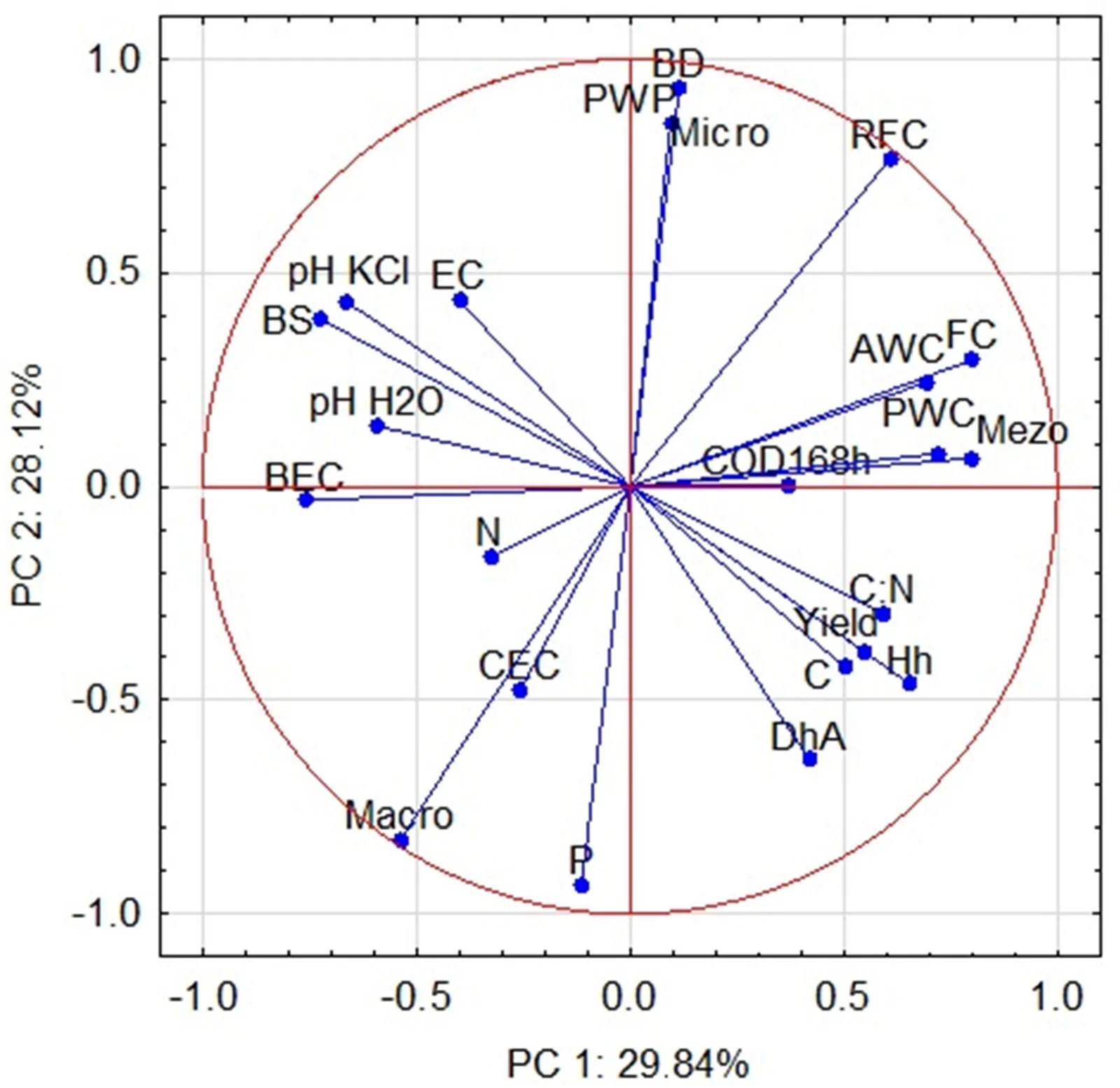
PCA loading plot for soil physical, chemical, and biological variables. The first two principal components explained 58.0% of the total variance.

**Figure 5:**
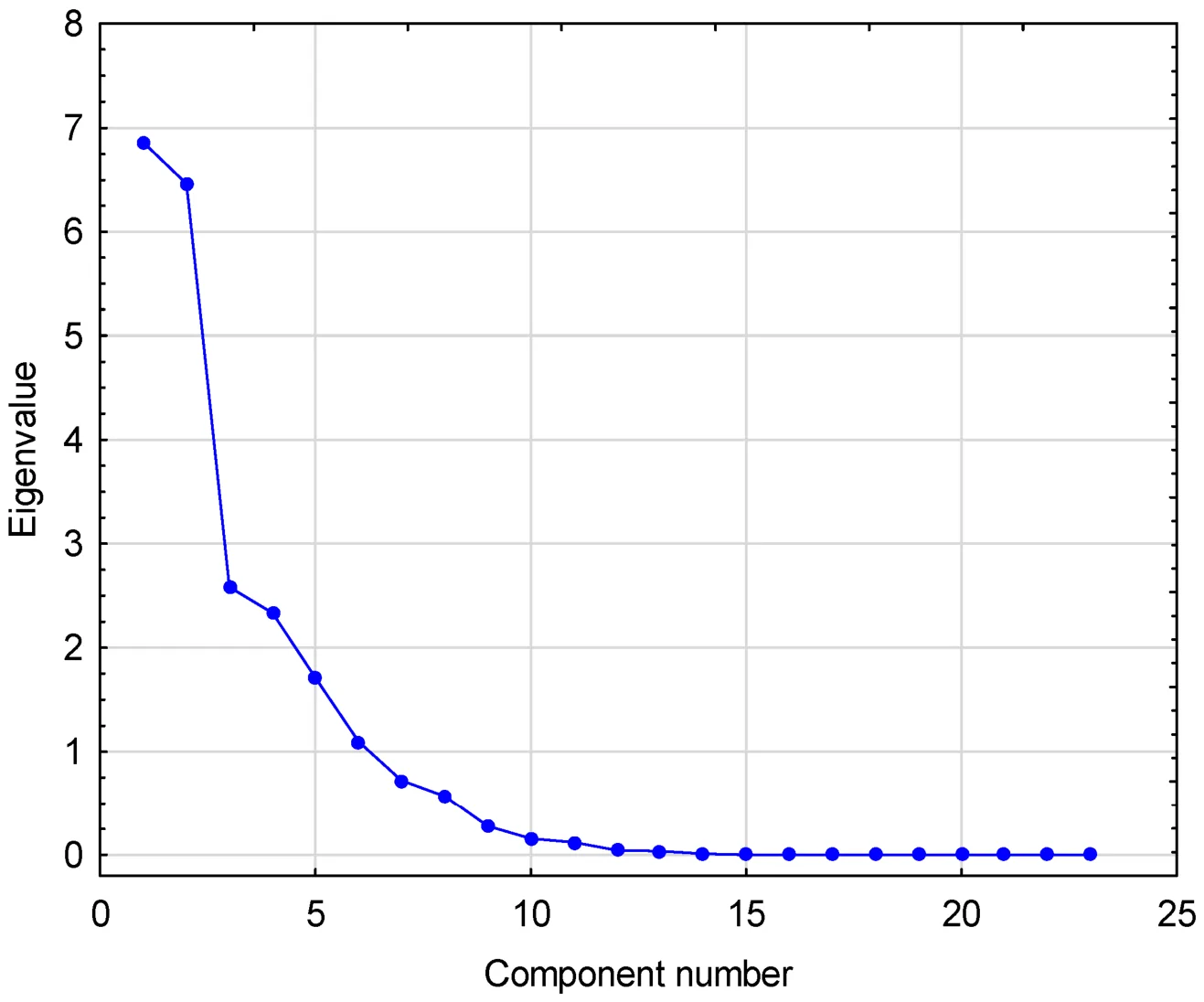
Scree plot of eigenvalues for the principal component analysis. The inflection after the second component supports retention of two components.

The first two principal components explained 58.0% of the total variance, with 29.84% explained by PC1 and 28.12% by PC2 (Figure 4 and Figure 5). PC1 was associated mainly with field capacity, available water content, productive water content, and mesoporosity, all of which had positive loadings ≥ 0.60. Exchangeable base cations, base saturation, and pH in KCl loaded negatively on PC1 (Table 6). PC2 was associated positively with bulk density, permanent wilting point, microporosity, and relative field capacity, and negatively with total porosity and macroporosity.

Dehydrogenase activity loaded negatively on PC2 (−0.640). Soil organic carbon and ryegrass biomass had loadings below the predefined |0.60| threshold and were therefore not used to define either component. The biplot summarizes the relationships among variables, whereas the scree plot supports retention of the first two components.

### 3.7. Water Retention Curve Modelling

The mean saturated water content parameter (θ_s_), fixed at total porosity for each pot, ranged from 0.506 cm^3^ cm^−3^ in MF+SSC to 0.540 cm^3^ cm^−3^ in MF+PL (Table 7). The mean α parameter ranged from 0.221 kPa^−1^ in MF+BC to 0.545 kPa^−1^ in CTR, with no significant pairwise differences according to Tukey’s HSD test. The n parameter varied within a narrow range, from 1.203 in CTR to 1.259 in MF. MF differed from CTR, whereas the remaining treatments were intermediate. Mean R^2^ ranged from 0.769 to 0.855 and mean RMSE from 0.0446 to 0.0588 cm^3^ cm^−3^. The highest mean R^2^ was obtained for MF+C(PL+BC), and the lowest mean RMSE for CTR. These fit statistics indicate appreciable departures between the constrained model and the measurements. The fitted α and n values therefore describe the curves conditional on the fixed θr and θs values and should not be treated as direct measurements of pore structure.

## 4. Discussion

The present study evaluated the effects of selected organic and mineral amendments and mineral fertilization on the amount of ryegrass biomass, selected chemical properties of the soil, biochemical activity, and soil physical quality, including pore size distribution and soil water retention characteristics. The observed functional changes in the soil, chemical, and biochemical properties and bulk density, porosity, and water retention parameters highlight the distinct and, in some cases, complementary ways in which organic and mineral amendments modify soil chemical, biochemical properties, structure, and hydraulic behavior.

The materials in Table 1 differed markedly in carbon content: 444 g kg^−1^ DM in BC, 280–302 g kg^−1^ DM in PL and the composts, and 1.9 g kg^−1^ DM in SSC. Together with their composition (Section 2.2), these differences provide context for the contrasting responses in storage pores and water retained at high suction. However, Table 1 reports chemical properties, so it does not directly establish differences in material porosity or surface area. The physical mechanisms discussed below remain possible explanations.

### 4.1. Chemical Parameters of Soil

The results of the present study were consistent with the well-known acidifying effect of mineral fertilization on soil. This effect is primarily due to the nitrification of N-NH_4_^+^ and the subsequent release of H^+^ ions into the soil solution. The results obtained by Guo et al. [32] indicate that the long-term use of nitrogen fertilizers in intensive farming systems leads to systematic soil acidification. Similar mechanisms were described by Bolan et al. [33], who pointed to a direct link between nitrogen transformations and changes in soil pH. The addition of biochar was found to partially limit soil acidification, consistent with previous literature. A meta-analysis by Jeffery et al. [34] showed that applying biochar often increased or stabilized pH, particularly in acidic and slightly acidic soils. This is usually explained by biochar’s alkaline nature and its ability to increase soil sorption capacity. O’Laughlin and McElligott [35] also emphasized that biochar can act as a buffering agent, mitigating the negative effects of mineral fertilizers’ acidifying action. Effects analogous to those witnessed in the biochar treatment with regard to the mitigation of the acidifying effects of mineral fertilization were obtained in treatments where smectite-silica clay and composts were applied in conjunction with NPK [36,37]. In the context of biochar and compost applications, the mitigating effect on soil pH is primarily attributable to the release of Ca^2+^ and Mg^2+^ ions into the soil solution. For smectite-silica clay soil amendments, the primary mechanisms were sorption, cation exchange, and clay mineral surface reactions [37].

The absence of significant differences in electrical conductivity (EC) among the treatments indicates that the applied doses of mineral salts and mineral–organic amendments did not result in a measurable increase in soil salinity under the experimental conditions. This finding is relevant because mineral fertilizers and organic amendments may modify soil EC through the addition and redistribution of soluble ions. However, the magnitude and direction of these changes depend strongly on the application rate, chemical composition and physicochemical properties of the amendment, as well as on the initial salinity status of the soil. Recent meta-analyses indicate that the effect of biochar on soil EC is not consistent across different soil and experimental conditions. For example, Su et al. [38] reported an overall 7.4% reduction in soil EC following biochar application to salt-affected soils, while the response was strongly regulated by initial soil salinity, biochar properties, and application rate. Similarly, Wu et al. [39] demonstrated that the effects of biochar on soil salinity varied according to biochar EC, application rate, pyrolysis temperature, and other experimental conditions. Field-scale evidence from alkaline soil also showed that biochar application did not significantly increase soil EC [40]. These findings support the interpretation that biochar and other organic amendments do not necessarily increase soil salinity, particularly when applied at moderate rates. Therefore, the lack of significant changes in EC observed in the present study suggests that the applied amendment rates did not impose an additional salinity burden on the soil and that the release of soluble constituents was insufficient to cause a detectable increase in soil EC during the experimental period.

Organic carbon content (C_org_) did not differ significantly between treatments, despite the introduction of carbon-rich materials. Similar observations in short-term experiments were reported by Major et al. [41], indicating that an increase in soil C content following biochar application may be difficult to detect in the first years of the study, particularly at moderate doses. A meta-analysis by Luo et al. [42] showed that a significant increase in C_org_ is more frequently observed in long-term studies. The lack of significant changes observed in the present experiment may therefore be related to the relatively short duration of soil exposure to the applied amendments.

A substantial increase in the total nitrogen (N_tot_) content of the soil in the MF+C(PL+BC+SSC) treatment indicates a positive response to the applied composite material, which included compost and a clay component. Six et al. [43] suggested that the stabilization of organic matter and nitrogen in association with clay minerals may reduce their mineralization and leaching. Stevenson [44] also highlighted the importance of the combined role of organic matter and the clay fraction in nitrogen retention in soil. In consideration of these findings, it can be assumed that the presence of smectite-silica clay promoted the reduction of nitrogen losses and its increased accumulation in the soil.

The C:N ratio remained at a level typical for soils with adequate nitrogen availability (approx. 8–10), indicating that mineralization processes predominated over immobilization. These values fall within the range considered optimal for soil microorganism activity [44]. The absence of significant differences in the C:N ratio among the soils of the individual treatments suggests that the applied materials did not disrupt the balance of organic matter transformation during the experiment.

### 4.2. Parameters of the Sorption Complex

An analysis of the sorption complex parameters revealed that the mixtures used significantly altered the soil’s chemical properties, particularly with regard to hydrolytic acidity (Hh) and the degree of saturation of the sorption complex with bases (V). The MF treatment had the highest Hh value, suggesting an acidifying effect of mineral fertilization, particularly nitrogen-based fertilizers. The sorption capacity of the complex remained relatively stable across all treatments; however, the lowest value was recorded in the MF+BC treatment and the highest in the MF+C(PL+BC+SSC) treatment. These results suggest that biochar alone does not significantly increase soil sorption capacity, possibly due to its properties (e.g., degree of aromaticity, specific surface area) or dosage [45,46,47]. In contrast, combining compost with smectite-silica clay favored maintaining a high T value, which can be explained by forming stable organic-mineral complexes. The presence of smectite-silica clay may have increased the number of sorption sites and stabilized organic matter in the soil [45]. Despite the increase in the Hh value in the MF soil, the total sorption capacity did not decrease significantly. This suggests that the changes primarily affected the composition of the sorption complex (the ratio of basic to acidic cations), rather than its overall size. The findings suggest that integrating mineral fertilization with organic and mineral materials could be a viable approach to enhance the sustainability of soil chemical properties [47,48,49].

### 4.3. Dehydrogenase Activity 

Dehydrogenase activity (DHA) is widely used as a sensitive indicator of soil biological and metabolic activity because dehydrogenases are intracellular enzymes involved in microbial redox processes and are closely associated with the metabolic state of the soil microbial community [50]. Recent studies have confirmed the usefulness of DHA for assessing changes in soil biological functioning and fertility in response to agricultural management practices, including organic amendments and fertilization [50,51,52]. However, DHA should be interpreted as an indicator of overall microbial metabolic activity rather than a direct measure of microbial viability, as its activity may also be influenced by soil physicochemical conditions, substrate availability, and environmental factors [51,52]. Thus, changes in DHA observed in the present study provide evidence of differences in the intensity of microbial biochemical activity among the applied treatments.

Our findings suggest that the MF treatment exhibited the highest level of dehydrogenase activity, thereby validating the hypothesis that the presence of readily available nutrients fosters the intensification of microbial metabolism [53]. Similar effects were reported in the study by Liu et al. [49], in which the application of mineral fertilizers increased soil enzymatic activity by enhancing the availability of nitrogen and phosphorus. Organic amendments, such as biochar and poultry litter, moderately increased dehydrogenase activity compared to the control; however, these values were lower than those in the MF treatment. According to Agegnehu et al. [54], the presence of organic materials in soil has a positive impact on microbial activity. However, the efficacy of these materials is contingent upon the rate of mineralization and the availability of nutrients in the environment. In our study, the MF+SSC, MF+C(PL+BC), and MF+C(PL+BC+SSC) treatments exhibited lower, more variable activity. This may result from slower nutrient release or an adverse effect of mineral fractions on soil microorganisms [48]. The variable response of microorganisms may result from differences in soil structure and the physicochemical properties of the applied amendments. In particular, factors such as pH, nutrient availability, organic carbon content, porosity, surface area, and water-holding capacity can modify microbial habitat conditions and substrate availability. These properties influence soil aeration, moisture retention, nutrient dynamics, and the accessibility of organic substrates, thereby affecting microbial growth and enzymatic activity [46]. Consequently, the effects of organic and mineral amendments on soil enzymatic activity depend not only on the characteristics of the amendments themselves but also on interactions with soil properties and environmental conditions. Combining mineral fertilizers with appropriately selected organic materials may therefore promote sustained soil biological activity by improving both nutrient supply and the physical environment for microorganisms.

### 4.4. Respiratory Activity

Soil respiratory activity is a complex parameter influenced by numerous biotic and abiotic factors. It serves as a measure of the overall level of soil metabolism. The efficacy of this parameter is contingent upon the implementation of agronomic practices, including the application of organic matter, mineral fertilizers, and soil conditioners to the soil [55,56]. An increase in respiration is generally caused by increased porosity, which increases the availability of oxygen to microorganisms. It is also caused by the direct and indirect effects of roots. In our study, higher respiratory activity was observed in the MF+SSC and MF+PL treatments, indicating enhanced microbial metabolism and carbon turnover. This observation aligns with the extant literature, which indicates that amendments containing readily degradable organic fractions can lead to a short-term increase in respiratory activity through a “priming” effect (stimulation of organic matter decomposition) [57]. In contrast, lower values of cumulative oxygen consumption in treatments containing biochar (particularly MF+C(PL+BC)) may be consistent with mechanisms reported in the literature whereby biochar reduces the accessibility of organic substrates and modifies microbial activity. However, because respiration measurements do not distinguish between amendment-derived carbon and native soil organic carbon, the present results cannot be interpreted as direct evidence of organic matter stabilization or carbon sequestration. This mechanism is well-documented in the literature. Biochar increases the proportion of stable carbon fractions and reduces the activity of microorganisms that decompose readily available organic compounds. Consequently, biochar may alter microbial activity and carbon turnover dynamics in soil, although the extent to which these processes contribute to long-term carbon stabilization cannot be determined from respiration measurements alone. However, it should be noted that the effect of biochar on soil respiration is ambiguous. Some studies showed no significant effect on basal respiration [58], while others indicated stimulating and inhibitory effects depending on the dose, soil properties, and environmental conditions [59]. In the long term, biochar may decrease respiration by several percent, which has been interpreted in the literature as being consistent with mechanisms that promote the protection and persistence of organic carbon in soil [60]. Intermediate values of soil respiratory activity were observed in the MF+C(PL+BC+SSC) treatment, which may reflect the combined characteristics of the applied composite material. Easily biodegradable materials increase microbial activity, while stable fractions (e.g., biochar) may limit these processes’ rates through the sorption of organic compounds and changes in substrate availability. Statistically significant differences between the treatments indicate that the selection of mineral and organic materials may influence the level and dynamics of biological soil processes. While high respiratory activity may indicate intense microbial activity, it is not always beneficial for long-term soil fertility as it can lead to accelerated mineralization and carbon loss. The results of this study indicate that soil biological activity may be influenced by the selection of appropriate amendments. This finding may have implications for plant productivity and soil carbon cycling; however, longer-term studies are required to evaluate potential effects on soil carbon stabilization.

### 4.5. Biochar-Induced Changes in Soil Structure, Soil Water Retention and Soil Physical Quality

MF+BC had higher field capacity, available water content, productive water content, and relative field capacity than CTR, whereas its differences from mineral fertilization alone were not significant. These results are consistent with previous studies indicating that biochar application may enhance soil water-holding capacity, particularly in coarse-textured or structurally degraded soils [61,62,63]. The improvement in FC and AWC is primarily attributed to the highly porous structure of biochar, which introduces additional pore space across a wide range of pore diameters and increases the soil’s specific surface area [64,65].

The increased volume of storage pores within the 0.005–0.5 μm and 0.5–50 μm diameter classes observed in MF+BC supports previous findings that biochar shifts pore size distribution toward pore classes that are most effective for plant water availability [66,67]. Mesopores are particularly important because they retain water at matric potentials accessible to plant roots, while still allowing adequate aeration [68]. The steeper slope at the inflection point of the soil water retention curve (S) describes a difference in water release [65,69]. It does not directly measure pore connectivity or aeration, which were not determined in this study.

Bulk density did not differ between MF+BC and CTR, indicating that improved water retention was not accompanied by an increase in bulk density. Similar observations have been reported in field and laboratory studies, where biochar improved soil water retention without adversely affecting bulk density or penetration resistance when applied at moderate rates [63].

Soil amended with poultry litter showed relatively limited changes in soil physical parameters compared to MF+BC. Although organic manures are commonly reported to reduce bulk density and increase total porosity by enhancing soil aggregation [70,71], the magnitude of these effects depends strongly on application rate, organic matter quality, and time elapsed since incorporation. In the present study, the absence of significant differences in bulk density and AWC between MF+PL and CTR indicates that the poultry litter did not produce detectable changes in these properties during the experiment.

The relatively small numerical increases in AWC and PWC under MF+PL may reflect the low application rate (0.5%) and the short time available for changes in soil structure. Their persistence cannot be assessed from this experiment. In a two-year field study with cattle manure on sandy soil, Lebrun et al. [72] found that adding biochar helped maintain the initial increase in soil moisture, whereas the effect of manure alone declined. Skadell et al. [73] reported only a small average increase in plant-available water capacity after long-term farmyard manure application. These findings suggest that the response depends on material type, soil conditions, and duration. They do not establish the mechanism responsible for the response to poultry litter in this study.

The two compost treatments produced intermediate physical responses. MF+C(PL+BC) had a relatively large volume of storage pores, but its available water content did not differ significantly from CTR. MF+C(PL+BC+SSC) had available water content comparable to MF and MF+BC without the increase in bulk density observed in MF+SSC. Composting can alter biochar surfaces and stabilize organic matter, which may explain why compost mixtures differ from the corresponding raw materials [54]. These results do not show a consistent advantage of the composts over all individual amendments. The intermediate responses are consistent with differences in compost composition and processing, but do not establish synergy between the components. Longer experiments are needed to determine whether these intermediate responses persist or change as compost-derived organic matter interacts with soil aggregates.

The soil treated with smectite-silica clay was characterized by increased bulk density and higher permanent wilting point, consistent with a shift toward finer pore fractions. The surface area and water adsorption properties of clay minerals may contribute to this response [72]. These pores retain water at high matric potentials, contributing to increased PWP, but not necessarily improving plant-available water.

Clay amendments can alter water adsorption and the packing of soil particles, with effects that depend on soil texture [72,73]. The reduced total porosity observed in MF+SSC may reflect changes in particle packing. Gas exchange and resistance to root penetration were not measured, so their responses cannot be established from these results.

MF+C(PL+BC+SSC) had higher AWC than CTR, without the increase in bulk density observed in MF+SSC. This contrast suggests that compost composition may modify the physical response to clay addition. Organic matter can promote aggregate formation and stabilize macropores, which may influence the response to clay addition [74]. However, equal total amendment rates supplied less clay in the compost treatment than in MF+SSC. The contrast therefore cannot be attributed solely to an interaction between clay and organic matter.

The higher AWC and RFC observed for MF+C(PL+BC+SSC) indicate that this treatment effectively increased the proportion of pores within the plant-available range. Integrated organic–mineral systems have been shown to optimize soil pore networks by combining adsorption-dominated micropores with structurally stable mesopores. RFC did not differ significantly between these treatments. Xuan et al. [75] assessed pore connectivity directly by X-ray microtomography in waterlogged soil. Such measurements were not made here. The present results support further evaluation of the com-post, but do not demonstrate improved pore connectivity or resilience under field conditions.

### 4.6. Implications for Soil Management

From a soil management perspective, the improvements in water retention observed in MF+BC and compost-based treatments are particularly relevant in the context of increasing climatic variability and water scarcity. Enhanced AWC and PWC can improve crop resilience to short-term drought by prolonging soil moisture availability between rainfall or irrigation events [45,61]. Drought tolerance was not tested because the pots were watered regularly. However, the results also indicate that not all amendments uniformly improve soil physical quality, suggesting the need for site-specific amendment strategies.

The findings suggest that biochar and some compost treatments can improve soil water retention under the conditions of this experiment. Long-term field studies are required to evaluate the persistence of these effects under different tillage practices, crop rotations, and climatic conditions.

### 4.7. Ryegrass Biomass

The results of the study indicate that mineral fertilization significantly increased plant biomass compared with the unfertilized control. This finding indicates that the availability of readily available NPK forms may play an important role in plant growth and development and, consequently, in biomass production. Concurrently, previous studies indicate that combining mineral fertilization with organic materials may improve nutrient use efficiency and soil properties [54]. In the present study, the highest amount of biomass was obtained where poultry litter was applied in addition to mineral fertilization (MF+PL). This can be explained by the high concentration of available nitrogen and the relatively fast mineralization rate of this material. Similar results were obtained by Agegnehu et al. [54], who reported that the combined application of mineral fertilization and organic materials may have beneficial effects on crop yield. Liu et al. [76] also indicated that the introduction of nitrogen into the soil in the form of organic materials, particularly in combination with NPK, had a beneficial effect on plant productivity as a result of increased nitrogen availability. The increase in the pool of available nitrogen forms may result from heightened soil microbial activity, thereby intensifying mineralization processes, which is primarily attributed to the application of organic materials [76]. The application of biochar in combination with mineral fertilization (MF+BC) resulted in biomass yields that were comparable to those obtained in the MF treatment. According to the meta-analysis by Ye et al. [48], the effect of biochar on plant biomass is strongly related to soil type, application rate, and experiment duration. Under short-term conditions, the effect of biochar may be difficult to detect. Similarly, Jin et al. [77] indicated that the positive effects of biochar are more evident in the long term due to improved soil structure and nutrient retention. Treatments where biochar-litter-based composts were applied had lower amounts of biomass removed compared to treatments where only biochar was added to the soil. These results support the hypothesis that the fertilizing effects of composts are long-term and manifest as improved soil properties and stabilized nutrients over time. The lowest ryegrass biomass was obtained in treatments where fertilization was applied and smectite-silica clay was introduced into the soil alongside mineral fertilization (MF+SSC). This may suggest the temporary immobilization of nutrients. Clay minerals increase the retention of ammonium and potassium ions, but they may also temporarily limit their availability to plants. Zhang et al. [45] showed that the effects of adding a clay fraction to the soil depend on the balance between the sorption and release of nutrients.

## 5. Conclusions

The findings of the study revealed that the use of amendments with disparate degradability capabilities enables the concurrent regulation of microbial activity and soil physical properties. Respiration analysis indicated that the MF and MF+SSC treatments exhibited the highest oxygen consumption, suggesting enhanced microbial activity and carbon turnover. In the case of MF+SSC, this response was most likely related to indirect effects on soil physicochemical conditions and substrate availability rather than the supply of readily degradable carbon. In contrast, the addition of biochar reduced respiratory activity (MF+C(PL+BC): 1.356 mg O_2_ g^−1^ DM), indicating lower oxygen consumption and altered microbial activity. This pattern is consistent with stabilization mechanisms reported in the literature; however, the respiration measurements used in this study do not provide direct evidence of organic matter stabilization or carbon sequestration. The first two principal components accounted for 58% of the variance and suggested associations between soil water properties, pore structure, and biological activity. These associations are exploratory. PC1 was associated mainly with water retention (FC, AWC, PWC), while PC2 reflected soil structural properties (BD, porosity). Biochar increased field capacity and available water content, whereas θ_s_ in the van Genuchten model was fixed at total porosity and was not an independently fitted measure of improvement. In contrast, the addition of smectite-silica clay increased the proportion of micropores and water retention at more negative matric potentials, with lower total porosity. Aeration was not measured directly. The MF+BC and MF+C(PL+BC+SSC) treatments produced the most favorable soil functional properties, combining improved water retention with moderate biological activity and limiting the excessive mineralization of organic matter. These treatments provided the most balanced effect between biological and physical processes. The present study indicated that the selection of organic–mineral amendments may influence soil functional quality through changes in both microbial activity and structural-hydrological properties. The study findings suggest a considerable application potential of the tested materials in agricultural and reclamation practices, particularly in the context of improving water management and soil functional quality. However, the potential effects of these materials on long-term carbon stabilization and sequestration require confirmation under long-term field conditions. The conclusions on soil physical properties are limited to a pot experiment with one soil type. The persistence of the water retention effects requires confirmation under field conditions.

## Figures and Tables

**Table 1:** Selected chemical properties of the materials used in the study.

Parameter		PL	BC	SSC	C(PL+BC)	C(PL+BC+SSC)
DM	g kg^−1^	862	948	965	442	436
N		27.29	6.86	0.89	16.14	15.18
C		280	444	1.9	285	302
pH	-	6.63	7.52	6.82	6.68	6.66
EC	µS cm^−1^	3800	220	60	7960	7480
Cd	mg kg^−1^	0.74	0.29	0.13	0.56	0.62
Cu		18.38	9.76	68.13	15.19	16.54
Pb		7.88	1.55	7.1	5.42	6.62
Zn		197.8	88.1	43.7	157.4	169.1

DM—dry matter, EC—electrical conductivity.

**Table 2:** Chemical quality parameters of soil and parameters of the sorption complex after different amendment treatments.

Treatment	pH	EC µS cm^−1^	C_org_ g kg^−1^ DM	N_tot_ g kg^−1^ DM
CTR	7.25 c *	187 a	12.4 a	1.49 a
MF	6.95 a	128 a	14.5 a	1.44 a
MF+BC	7.09 b	129 a	14.1 a	1.42 a
MF+PL	7.00 ab	186 a	14.6 a	1.46 a
MF+SSC	7.00 ab	171 a	12.2 a	1.41 a
MF+C(PL+BC)	7.00 ab	186 a	13.0 a	1.47 a
MF+C(PL+BC+SSC)	6.94 a	191 a	13.7 a	1.64 b
**Treatment**	**S**	**Hh**	**T**	**V**
	**mmol(+) kg^−1^ DM**			**%**
CTR	96.9 c *	15.1 a	112.0 b	86.6 b
MF	91.4 ab	22.1 c	113.5 b	80.5 a
MF+BC	88.9 a	19.2 b	108.0 a	82.3 a
MF+PL	92.5 ab	20.2 bc	112.7 b	82.1 a
MF+SSC	91.6 ab	20.3 bc	111.8 ab	81.9 a
MF+C(PL+BC)	93.5 bc	19.4 bc	112.9 b	82.8 a
MF+C(PL+BC+SSC)	93.0 abc	21.2 bc	114.3 b	81.4 a

* Different letters in columns indicate a significant difference at *p* ≤ 0.05 according to Tukey’s HSD test. S—the sum of basic cations, Hh—hydrolytic acidity, T—total sorption capacity, V—the degree of saturation of the sorption complex with bases.

**Table 3:** Trend lines of changes in respiratory activity and cumulative oxygen demand of soil after different amendment treatments.

Treatment	Equation of the 1° Activity Curve (RA) During the Incubation Period of 24–168 h y = ax + b (*) (mg O_2_ g^−1^ DM)		Cumulative Oxygen Demand in 168 h (mg O_2_ g^−1^ DM)
CTR	y = 0.0105x − 0.1879	R^2^ = 0.989	1.575 ab **
MF	y = 0.0118x + 0.1263	R^2^ = 0.988	1.787 ab
MF+BC	y = 0.0098x + 0.1122	R^2^ = 0.993	1.714 ab
MF+PL	y = 0.0111x + 0.0912	R^2^ = 0.993	1.734 ab
MF+SSC	y = 0.0129x + 0.2816	R^2^ = 0.994	1.805 a
MF+C(PL+BC)	y = 0.0093x + 0.1643	R^2^ = 0.987	1.356 b
MF+C(PL+BC+SSC)	y = 0.0092x + 0.0184	R^2^ = 0.955	1.456 ab

* x—hour; ** Different letters indicate a significant difference at *p* ≤ 0.05 according to Tukey’s HSD test. CTR—control soil; MF—soil with mineral fertilizers, MF+BC—soil with MF and biochar; MF+PL—soil with MF and poultry manure; MF+SSC—soil with MF and smectite–silica clay; MF+C(PL+BC)—soil with MF and compost made from poultry manure and biochar; MF+C(PL+BC+SSC)—soil with MF and compost made from poultry manure and biochar, with the addition of smectite–silica clay.

**Table 4:** Physical quality parameters of soil after different amendment treatments.

Treatment **	BD (g cm^−3^)	FC (cm^3^ cm^−3^)	PWP (cm^3^ cm^−3^)	AWC (cm^3^ cm^−3^)	PWC (cm^3^ cm^−3^)	S	RFC
CTR	1.253 a *	0.356 b	0.117 ab	0.234 b	0.208 b	0.0651 ab	0.676 b
MF	1.240 a	0.376 ab	0.115 ab	0.260 a	0.234 a	0.0702 ab	0.706 ab
MF+BC	1.252 a	0.385 a	0.114 ab	0.265 a	0.236 a	0.0737 a	0.730 a
MF+PL	1.220 a	0.365 ab	0.111 b	0.238 ab	0.213 ab	0.0693 ab	0.679 b
MF+SSC	1.309 b	0.368 ab	0.120 a	0.247 ab	0.218 ab	0.0645 b	0.731 a
MF+C(PL+BC)	1.254 a	0.373 ab	0.113 ab	0.247 ab	0.222 ab	0.0718 ab	0.709 ab
MF+C(PL+BC+SSC)	1.235 a	0.370 ab	0.112 ab	0.260 a	0.234 ab	0.0672 ab	0.694 ab

* Different letters in columns indicate a significant difference at *p* ≤ 0.05 according to Tukey’s HSD test; ** BD—bulk density, FC—field capacity, PWP—permanent wilting point, AWC—available water content, PWC—productive water content, S—slope at the inflection point of water retention curve, RFC—relative field capacity. CTR—control soil; MF—soil with mineral fertilizers; MF+BC—soil with MF and biochar; MF+PL—soil with MF and poultry manure; MF+SSC—soil with MF and smectite–silica clay; MF+C(PL+BC)—soil with MF and compost made from poultry manure and biochar; MF+C(PL+BC+SSC)—soil with MF and compost made from poultry manure and biochar, with the addition of smectite–silica clay.

**Table 5:** Pore size distribution of the soil after different amendment treatments.

Treatment	Volume of Pores (cm^3^ cm^−3^) for Diameter Classes (μm)					TP ** (cm^3^cm^−3^)
	<0.005	0.005–0.5	0.5–50	50–500	>500	
CTR	0.070 a *	0.102 ab	0.240 b	0.099 a	0.017 a	0.527 a
MF	0.064 ab	0.108 ab	0.265 ab	0.084 a	0.011 a	0.532 a
MF+BC	0.059 b	0.113 a	0.282 a	0.066 a	0.007 a	0.527 a
MF+PL	0.064 ab	0.102 b	0.251 ab	0.105 a	0.018 a	0.540 a
MF+SSC	0.069 a	0.108 ab	0.249 ab	0.070 a	0.009 a	0.506 b
MF+C(PL+BC)	0.059 b	0.107 ab	0.271 ab	0.080 a	0.010 a	0.527 a
MF+C(PL+BC+SSC)	0.069 a	0.105 ab	0.250 ab	0.095 a	0.015 a	0.534 a

* Different letters indicate a significant difference at *p* ≤ 0.05 according to Tukey’s HSD test. ** TP—total porosity. CTR—control soil; MF—soil with mineral fertilizers; MF+BC—soil with MF and biochar; MF+PL—soil with MF and poultry manure; MF+SSC—soil with MF and smectite–silica clay; MF+C(PL+BC)—soil with MF and compost made from poultry manure and biochar; MF+C(PL+BC+SSC)—soil with MF and compost made from poultry manure and biochar, with the addition of smectite–silica clay.

**Table 6:** Loadings of variables on principal components.

Parameter	PC 1	PC 2
BD	0.115	**0.933**
FC	**0.800**	0.297
PWP	0.095	**0.852**
P	−0.115	**−0.933**
PWC	**0.720**	0.077
AWC	**0.693**	0.244
RFC	**0.608**	**0.768**
Macro	−0.538	**−0.827**
Mezo	**0.797**	0.067
Micro	0.095	**0.852**
pH KCl	**−0.666**	0.432
pH H_2_O	−0.593	0.142
EC	−0.401	0.436
DhA	0.419	**−0.640**
C	0.503	−0.422
N	−0.328	−0.164
C:N	0.593	−0.298
Yield	0.550	−0.388
Hh	**0.654**	−0.461
BEC	**−0.762**	−0.032
CEC	−0.257	−0.474
BS	**−0.729**	0.394
COD168h	0.369	0.006

Values ≥ |0.60| are highlighted in bold.

**Table 7:** Parameters of the three-parameter van Genuchten model and goodness-of-fit statistics for soil water retention curves.

Treatment	*θs*	*α* (kPa^−1^)	*n*	R^2^	RMSE
CTR	0.527 ± 0.023 a *	0.545 ± 0.185 a	1.203 ± 0.009 b	0.837 ± 0.050	0.0446 ± 0.0090
MF	0.531 ± 0.018 a	0.271 ± 0.231 a	1.259 ± 0.056 a	0.821 ± 0.058	0.0518 ± 0.0061
MF+BC	0.527 ± 0.025 a	0.221 ± 0.116 a	1.250 ± 0.032 ab	0.819 ± 0.025	0.0548 ± 0.0021
MF+PL	0.540 ± 0.026 a	0.458 ± 0.327 a	1.236 ± 0.032 ab	0.852 ± 0.034	0.0448 ± 0.0061
MF+SSC	0.506 ± 0.030 b	0.317 ± 0.336 a	1.246 ± 0.042 ab	0.815 ± 0.029	0.0498 ± 0.0054
MF+C(PL+BC)	0.527 ± 0.027 a	0.269 ± 0.104 a	1.242 ± 0.012 ab	0.855 ± 0.029	0.0469 ± 0.0050
MF+C(PL+BC+SSC)	0.534 ± 0.021 a	0.383 ± 0.188 a	1.230 ± 0.041 ab	0.769 ± 0.022	0.0588 ± 0.0029

Values are means ± SD. θ_r_ was fixed at 0 cm^3^ cm^−3^, θ_s_ was fixed at total porosity, α and n were fitted, and m was calculated as 1 − 1/n. * Different letters indicate significant differences according to Tukey’s HSD test (*p* ≤ 0.05). CTR—control soil; MF—soil with mineral fertilizers; MF+BC—soil with MF and biochar; MF+PL—soil with MF and poultry manure; MF+SSC—soil with MF and smectite–silica clay; MF+C(PL+BC)—soil with MF and compost made from poultry manure and biochar; MF+C(PL+BC+SSC)—soil with MF and compost made from poultry manure and biochar, with the addition of smectite–silica clay.

## Data Availability

The original contributions presented in this study are included in the article/Supplementary Materials. Further inquiries can be directed to the corresponding author.

## Supplementary Materials

The following supporting information can be downloaded at: https://www.mdpi.com/article/10.3390/ma19183916/s1, Table S1. One-way ANOVA for soil physical properties, water-retention variables, pore-size classes.
