# Supplementary material for: Biochar–Clay–Compost Composite Materials for Sustainable Soil Management: Effects on Soil Biochemical Activity, Pore Architecture, and Water Retention
Source: Materials (Basel). 2026 Sep 15;19(18):3916. doi: 10.3390/ma19183916 (PMC13608590; doi:10.3390/ma19183916)
Supplement: Supplementary file 1 [file materials-19-03916-s001.zip › materials-4511226-supplementary.pdf]

Table S1. One-way ANOVA for soil physical properties, water-retention variables, pore-size classes.

Variable	Unit	df	F	p
BD	g cm^{-3}	6	3.286	0.0282
FC	cm^3 cm^{-3}	6	3.309	0.0304
PWP	cm^3 cm^{-3}	6	5.471	0.0012
AWC	cm^3 cm^{-3}	6	4.987	0.0004
PWC	cm^3 cm^{-3}	6	5.810	0.0021
S	cm^3 cm^{-3}	6	3.147	0.0099
RFC	dimensionless	6	2.642	0.0327
Pores <0.005 μm	cm^3 cm^{-3}	6	2.757	0.0203
Pores 0.005–0.5 μm	cm^3 cm^{-3}	6	3.234	0.0085
Pores 0.5–50 μm	cm^3 cm^{-3}	6	4.109	0.0017
Pores 50–500 μm	cm^3 cm^{-3}	6	1.849	0.1061
Pores >500 μm	cm^3 cm^{-3}	6	1.058	0.1729
Total porosity	cm^3 cm^{-3}	6	2.286	0.0082

BD – bulk density, FC – field capacity, PWP – permanent wilting point, AWC – available water content, PWC – productive water content, S – slope at the inflection point of water retention curve, RFC – relative field capacity
